# Supplementary figures and images for: Prevalence and evolutionary analyses of human T-cell lymphotropic virus in Guangdong province, China: Transcontinental and Japanese subtype lineages dominate the prevalence
Source: PLoS Negl Trop Dis. 2021 Feb 4;15(2):e0009043. doi: 10.1371/journal.pntd.0009043 (PMC7888662; doi:10.1371/journal.pntd.0009043)

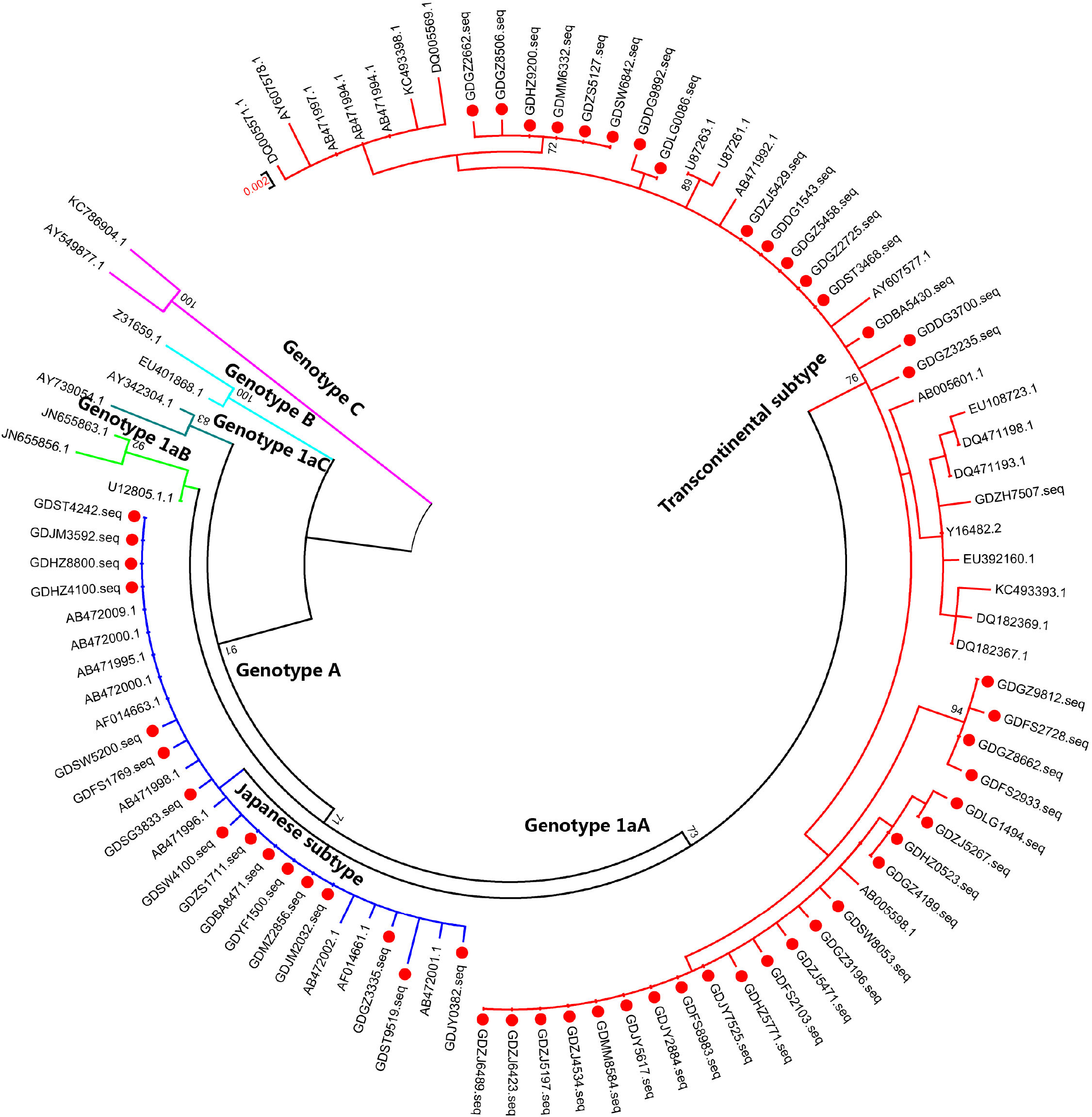

Supplement: S1 Fig — Support for the branching order was determined by 1000 bootstrap replicates; only values of 70% or more are shown. Red circles indicated sequences from blood donors in this study. (TIF) [file pntd.0009043.s001.tif]

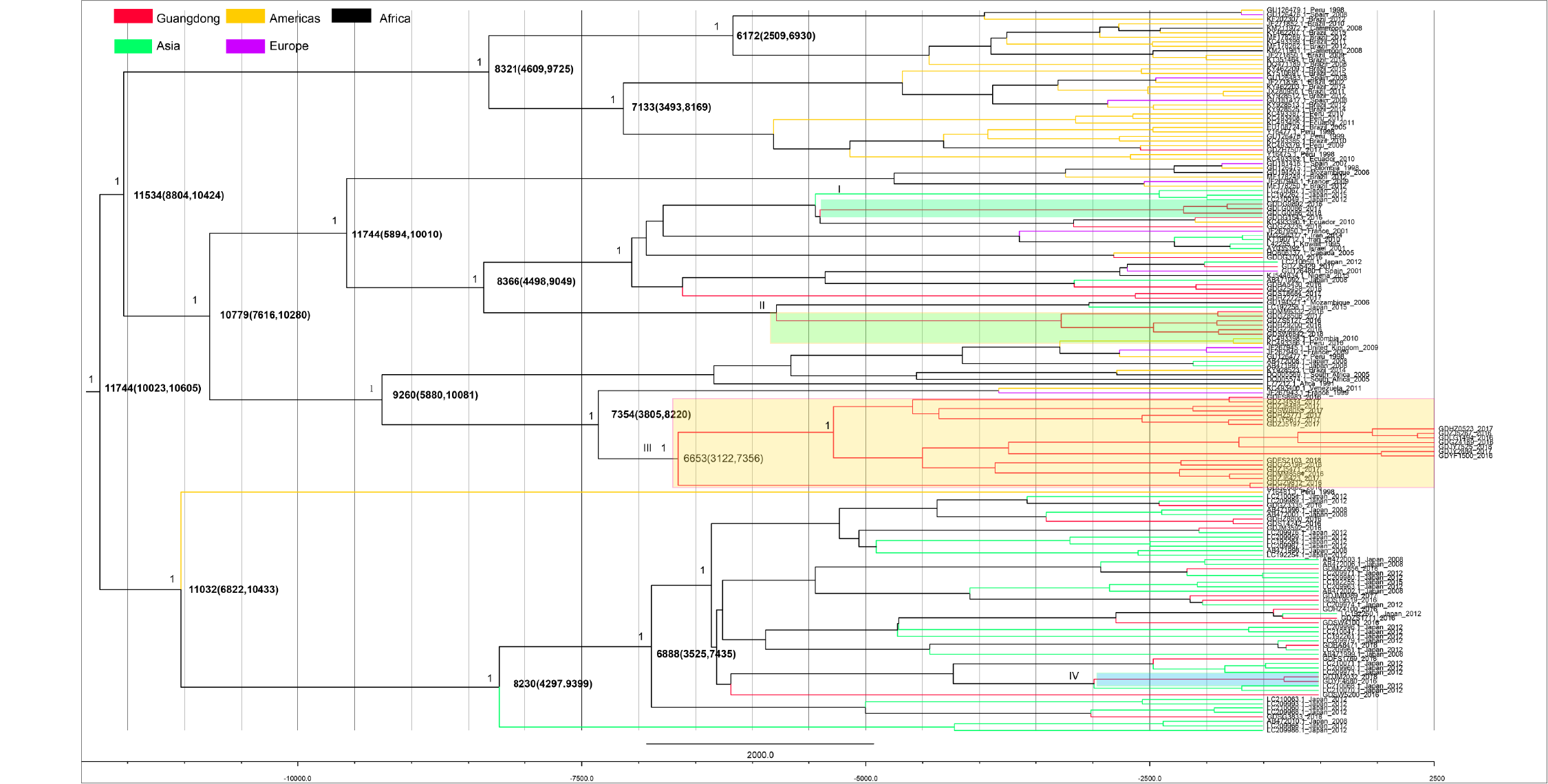

Supplement: S2 Fig — The names of sequences from this study and reference consist of the isolate name, province, country and time of specimen collection. Branches were colored according to their sampling regions in different continents or province. Time scale runs from 11744 years ago to 2018. Four clusters in the tree marked I, II, III and IV respectively showed the 4 different introduction events to Guangdong for HTLV-1. The posterior values of branches > 0.9 were shown only. (TIF) [file pntd.0009043.s002.tif]
