# Supplementary material for: Prevalence and evolutionary analyses of human T-cell lymphotropic virus in Guangdong province, China: Transcontinental and Japanese subtype lineages dominate the prevalence
Source: PLoS Negl Trop Dis. 2021 Feb 4;15(2):e0009043. doi: 10.1371/journal.pntd.0009043 (PMC7888662; doi:10.1371/journal.pntd.0009043)
Supplement: Attachment — Submitted filename: response.docx [file pntd.0009043.s003.docx]

Dear editors and reviewers,

Thank you for handling and reviewing our manuscript (PNTD-D-20-00867). We have revised the manuscript carefully and performed the phylogenetic analysis in multiple models suggested by the reviewers. Our responses to the questions and comments raised by the reviewers are as follow:

Reviewers’ comments:

Reviewer #1: The molecular clock has been used on gp46 with limited caution. It chould be completed with a study on other viral sequences (LTR) and the ESS for each node should be presented.
The legends of the phylogenetic trees that are presented lack information. Many more sequences available on genebank should be added.
**Response:** Thank you for your insightful comments. The LTR gene MCC tree has been constructed according to your suggestion (S2 Fig).

As supplemented in the methods section (page6 line 130-134, highlighted in yellow), according to the manual of BEAST software, when all ESS numbers were ≥200 sufficient sampling was considered to have been achieved.

The legends of both gp46 (Fig 3) and LTR (S2 Fig) phylogenetic tree were updated by adding related information.

Before conducting beast analysis, almost all the global representative reference sequences of similar length from Genbank were retrieved and downloaded. In order to minimizing computing time, identical sequences and sequences with insufficient information (e.g. acquisition time) for constructing MEGA trees were filtered out. The remaining sequences, which can represented all the available sequences, were used to construct MCC tree.

Reviewer #1: While the epidemiological aspect of the study seems sound, the part concerning datation is very insufficient.
**Response:** Thank you for your comments. The information including datation has been supplemented in legends of both gp46 (Fig 3) and LTR (S2 Fig) phylogenetic tree. All the changes have been highlighted in yellow for your reference.

Reviewer #1: There are different types of conclusions that are presented :
- the region has some HTLV, however can a region with 1/100.000 be considered as endemic?

**Response:** Thank you for your comments. We agreed with you that the prevalence of HTLV in Guangdong is generally low. In the manuscript the conclusion “endemic” has now replaced with “prevalence”.

- the datations are conclusions on movement of population considers that the transcontinental virus had been introduced only once in the region, which is not evident.
**Response:** Thank you for your comments. We realized that the description in the manuscript may not be clearly. Indeed, the phylogenetic analysis showed that HTLV-1A migrated to Guangdong and then formed four major clusters, excepted for some sporadic strains. Please see page2 line 35-38 for the revised description.

Reviewer #1: Authors have determined the seroprevalence of HTLV among blood donors in the Guangdong region, China. The conclusion is that the prevalence is low. Phylogenetic analyses demonstrate that circulating strains belong either to TC or Japanese sub clades. While the study is of interest, it is unclear whether the region can be considered as endemic (as the prevalence of very low) and datation methods and interpretations are very hazardous.
**Response:** Thank you for the comments. In China, HTLV is mainly prevalence in Guangdong, Fujian, and Zhejiang provinces. In our study, Guangdong is generally a low-prevalence area. The word “endemic” has now been replaced with “prevalence” in this manuscript. In addition, the datation methods have been updated, and MCC tree of LTR gene has been constructed to supplement more evidence (S2 Fig). The structures of gp46 and LTR gene MCC trees were similar.

Reviewer #2: Apart from some attention to the phylogenetic aspects of this work, the manuscript needs some language editing to correct for the many linguistic errors. For example, abstract, last sentence "reduce the risk of its infection" does not correctly word what is meant. There are many such issues throughout the manuscript, which at times make it unclear what exactly is meant by the authors.

**Response:** Thanks for the comment. The language of the manuscript has been improved by English editing service provided by Letpub.

Reviewer #2: I am not an expert on HTLV epidemiology, which is why I limit my comments to the phylogenetic aspects of this paper.

Abstract
=========

- "using Bayesian Markov chain Monte Carlo (MCMC) inference method." => to my knowledge, MCMC is always "Bayesian". Why not rephrase to "in a Bayesian phylogenetic framework" or something alike?

**Response:** Thank you for the comment. This phase has been replaced by “Bayesian phylogenetic framework”. Please see page1 line23-25 for the changes that is highlighted in yellow.

- "The phylogenetic analyses showed that HTLV-1A is spread into China (18202 years ago) and became endemic." => it is well possible that undersampling underlies the lack of apparent spread from China to Japan. The authors should rephrase references to this in a more nuanced manner.
**Response:** Thank you for the comments. The description has been revised following you suggestion (page2 line 35-38).

- 21 cities are mentioned in the abstract while HTLV positive samples were detected in only 16 cities (line 144)?
**Response:** Thank you for the comment. In this study HTLV antibody tests by ELISA and ECLIA were initially carried out in 21 cities. To exclude false-positive results, ELISA-reactive samples were then applied to confirmation experiments such as western blot (WB) and nucleic acid testing (NAT). Confirmed HTLV-positive samples were found in 16 cities, while the ELISA-reactive samples in the other five cities were all negative by NAT or WB.

Methods/results/discussion
==========================
- I am mostly concerned that with this combination of evolutionary rate and time scale, there can be a time-dependent effect on the evolutionary rate. As this can substantially impact the divergence datings, the authors should test for this by comparing the fit of a relaxed clock model (the model that was used by the authors) with that of a model that incorporates a time-dependent effect. For an example of this I refer to Membrebe et al, Mol Biol Evol 2019.
**Response:** Thank you for your insightful comments which have expanded our knowledge on phylogenetic analysis. Based on your suggestion, the relaxed clock model was compared with another model that incorporates a time-dependent. Please see page5 line 124-128 for the changes that is highlighted in yellow for your reference.

- how was the mixing of the chains evaluated?
**Response:** Thank you for your comments. Based on the structure of the phylogenetic tree, we think that during the transmission of the virus, some strains have evolved to form a dominant transmission strains with regional transmission characteristics, while other virus strains still retain the characteristics of shared virus strains to maintain sporadic transmission.

- did the authors use a skyline model, or a constant popsize model? There seems to be some ambiguity on this between the methods and results section.
**Response:** Thank you for the question. The skyline model was initially used in our analysis. Base on your suggestions, the time-dependent rate model with Markov jumps process was used, which showed a better Bayes factors and thus was written in the revised manuscript (page5 line 124-128).

- "18,202 years ago (95% CI: 22,340-89,339)" => how is it possible that the mean or median estimate does not lie within the 95% CI? This looks like an error (or typo?).
**Response:** My apology on the typing mistake. We have check for the analysis and corrected the number to “18,202 years ago (95% CI: 4,913-68,681)”. ( page8 line 183-185).

- "Phylogeographic tree analysis of HTLV-1A genotype revealed different
relatedness of the isolates from China and those from other countries" => what exactly is meant by this?

**Response:** I am sorry for the ambiguous writing. Actually we want to tell that some of our isolates are different from those in other countries, which indicating that such isolates may be unique and only found in China.

- an objective criterium should be used to classify strains as 'group A or B'. Which one was used?
**Response:** HTLV-1A contains three main subgroups: Transcontinental, Japanese and West African. Based on the structure of the tree, two branches (A and B) evolved from the root of the tree. According to the reference sequence information on the two branches, A was determined to be Transcontinental subtype, and B was Japanese subtype (see page8 line 186-188).

- "The major proportion of isolates from Guangdong were classified into clade I, II and III, which only contained isolates from China. Consequently, it is a China-specific clade. " => for such statements it is always good to keep in mind that undersampling can lead to a false image of population structure, or erroneous apparent migration links (see for example https://pubmed.ncbi.nlm.nih.gov/30118874/). I hence encourage the authors to make less strong statements when it comes to this.
Also, as it is not possbile to analyse a complete sample, the number of apparent introductions into China always will be a minimal estimate. As the authors seem to have overlooked that individual lineages can also represent introductions (fig 3), I suggest that they formally estimate the number of introductions into China using Markov jumps (can be set up in BAEUTi), of that they instead take a parsimonious approach to count the number of introduction events.
**Response:** Thank you for your insightful comments. We agreed that a solid conclusion cannot be fully supported by the current evidence, thus this part has been revised according to your suggestions. Please find the related content in the manuscript (page8 line188-195, and page11 line 263-268). All the changes has been highlighted in yellow for your reference.

Based on your second suggestion, Markov jumps process was used to formally estimate the number of introductions into China. The analysis result showed that fitting Markov jumps at the same time, time-dependent rate model is better than a relaxed clock model base on Bayes factors. Thus it has been finally adopted in the manuscript (see fig 3 and page8, line 179-183).

- it would increase the ease of interpretation of results if the authors could also indicate the genotype A subtypes (transcontinental etc) and the type of amino acids at gp46 positions 19 and 55 in the MCC or ML tree. A nice visualisation tool to this end is iTol (https://itol.embl.de/). The phylogenies should also be annotated with branch support.

**Response:** Thank you for your insightful comments. We used iTol to edit the phylogenetic tree as you suggested. It took some time since it is my first time using this tool. The phylogenetic tree was now annotated with branch support. Please see the updated fig 2 and fig 4.

-"Due to the genomic stability of HTLV-1," => what do the authors mean?
**Response:** What we mean by this sentence is that the gene mutation rate of HTLV is low (see page11 line 261-263), probably because of the low replication or inactivation of the virus in the host cells.

- "The communications between China and Japan are frequent and expatriates in China from Japanese enterprises are mostly in Guangdong." => the authors should discuss whether or not for HTLV the evolutionary and epidemiological events occur on the same time scale. For example, one could argue that given the 'deep' timing of the relevant branch(es) in the phylogeny, the explanation for the migration links should be historical rather than contemporaneous.

**Response:** Thank you for your insightful comments. Please see page11 line 268-274 for the revision. We have link the time nodes of the virus introduced into Guangdong China to the historical inter-communication of the two countries back to Han Dynasty.
