# Supplementary material for: Prevalence and evolutionary analyses of human T-cell lymphotropic virus in Guangdong province, China: Transcontinental and Japanese subtype lineages dominate the prevalence
Source: PLoS Negl Trop Dis. 2021 Feb 4;15(2):e0009043. doi: 10.1371/journal.pntd.0009043 (PMC7888662; doi:10.1371/journal.pntd.0009043)
Supplement: Attachment — Submitted filename: response.docx [file pntd.0009043.s004.docx]

Dear editors and reviewers,

Thank you for handling and reviewing our manuscript (PNTD-D-20-00867). We have revised the manuscript carefully and performed the phylogenetic analysis in 3rd codon position nucleotide substitution model suggested by the reviewers. Our responses to the questions and comments raised by the reviewers are as follow:

Reviewers’ comments:

Reviewer #1: When considering molecular clock on an encoding region (i.e. Env), authors should perform the analysis on the 3rd codon (there is indeed a different evolution rate depending on codons, the 3rd seem to be more reliable).

Considering PCR, authors should present the technique (nb of cycles, primer sequence).

**Response:** Thank you for your insightful comments. The gp46 (Fig 3) and LTR (Fig S2) phylogenetic trees have been constructed using 3rd codon position nucleotide substitution model according to your suggestion. Please see page 6 line 140-144, which has been highlighted in yellow for your reference.

The information for the PCR technique used in the current manuscript has been supplemented in the methods section (page5 line 108-125, highlighted in yellow).

Reviewer #1: It is well known that HTLV infection occurs on transfusion, but only is leucoreduction does not occur. Do blood banks in China perform leucoreduction? Please discuss

**Response:** Thank you for the comment. As far as we know, about one-third of blood banks in China have carried out leukoreduction, while only 5 out of 21 blood banks in Guangdong province have carried out this technology. The risk of HTLV transmission via blood transfusion cannot be completely eliminated by the current implementation of leukoreduction. Thus, HTLV screening in BDs is still an important risk mitigation strategy. Please see page13 line 318-321 in the discussion section for the changes that is highlighted in yellow for your reference.

Reviewer #1: Authors should note the subtypes: HTLV-1a, -1b, 1c and not HTLV-1A, 1-B, 1-C. (abstract, and lines 171:172)

**Response:** Thank you for pointing out my mistakes. We have checked and corrected the related depicts in the current manuscript.

Reviewer #1: The paper is interesting. For molecular clock, authors should add a study considering the 3rd codon of the coding sequence. Otherwise the manuscript is sound.

Please correct paraparesis (line 62), and prefer 'brest feeding' over 'breast milk' (line 65).

Line 72 is not clear: it is not because 'incubation period is long' that transfusion can occur. Please correct/

**Response:** Thank you for your insightful comments. The gp46 (Fig 3) and LTR (Fig S2) gene trees have been constructed using 3rd codon position nucleotide substitution model according to your suggestion. Compared with our original results, we found that gp46 gene is better with 3rd codon position nucleotide substitution model and LTR gene is better with 2nd codon position nucleotide substitution model (see page6 140-144, highlighted in yellow).

My apology for the language mistakes. We have corrected the relevant statements in this manuscript (line 62, 65 and 71-73, highlighted in yellow).
